# All-Laser Suite Analytical Platform (Microsampling, Catapulting, Trapping, and LIBS) for the Characterization of Discrete Domains in Samples of Astrochemical Interest

**DOI:** 10.1021/acs.analchem.6c01976

**Published:** 2026-08-28

**Authors:** Clara Burgos-Palop, Tomás Delgado, Pablo Purohit, Francisco J. Fortes, José M. Vadillo

**Affiliations:** † UMALASERLAB, Departamento de Química Analítica, 16752 Universidad de Málaga, 29071 Málaga, España, Spain; ‡ Instituto de Materiales y Nanotecnología (IMANA), Facultad de Ciencias, Universidad de Málaga, 29071 Málaga, España, Spain; § Universidad Complutense, Facultad de Químicas, Departamento de Química Analítica, 28040 Madrid, España, Spain

## Abstract

The chemical characterization of scarce or heterogeneous solid samples remains a significant analytical challenge due to the high material quantities required by conventional techniques. Here, a microsampling approach is presented that combines laser ablation, optical trapping, and single-particle laser-induced breakdown spectroscopy (μLA-OT-SP-LIBS) for the analysis of discrete microdomains with minimal sample consumption. In this scheme, particles were in situ generated by laser ablation from a geological target, the Jbilet Winselwan carbonaceous chondrite, then individually trapped using optical trapping, and subsequently analyzed by LIBS. A set of complementary techniques, including SEM-EDX, XRD, and XRF, was employed to characterize the particle morphology, its elemental composition, and the mineralogy of the bulk material. Spherical particles produced by LA exhibited low polydispersity, with average diameters of 1.31 and 1.46 μm for matrix- and chondrule-derived particles, respectively. Single-particle LIBS analysis enabled the discrimination of particles originating from Fe-rich matrix and chondrules based on characteristic intensity ratios (e.g., Mg/Fe, Na/Fe). Also, CN molecular bands were detected in some matrix-derived particles, thus indicating the presence of native organic carbon. In contrast, particles derived from the chondrule, a nonorganic domain, showed no detectable organic signal. These findings demonstrate that μLA-OT-SP-LIBS can successfully characterize exiguous, discrete domains with negligible sample damage and absolute mass requirements in the low microgram range. The proposed methodology provides a powerful platform for multielemental and molecular analysis of rare or valuable samples, with potential applications in meteoritics, geochemistry, and astrobiology.

## Graphical abstract

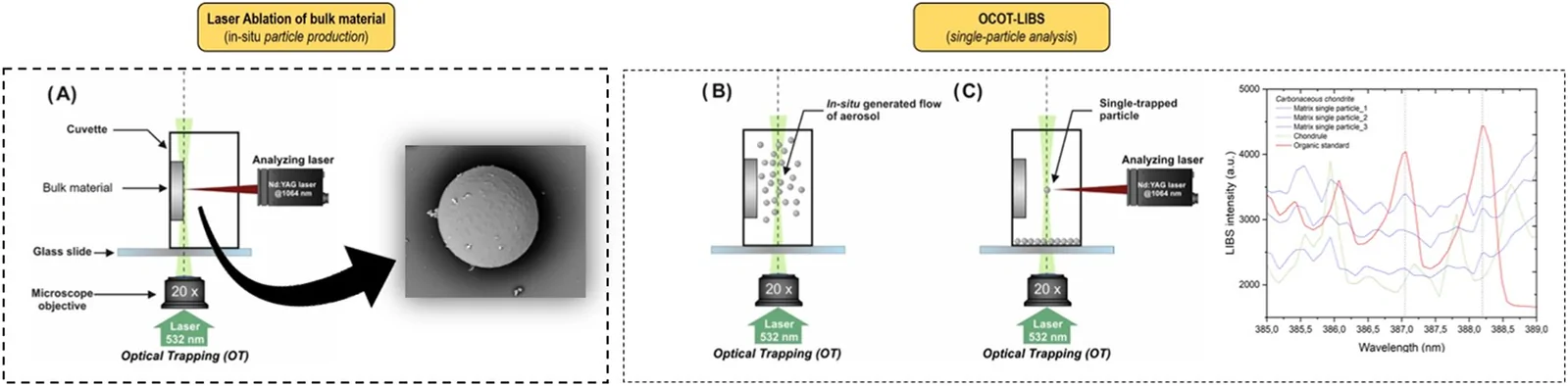

## Introduction

Scarce and/or valuable solid samples pose a significant challenge for conventional atomic analytical techniques, given the high absolute mass amounts they often require.^1^ Notwithstanding the phenomenal limits of detection (LOD) and quantification (LOQ) provided by well-established workhorses such as mass-spectrometry-based methods, sample quantities in the range of *mg* to *g* are required to perform a complete analysis even when combined with sophisticated sample preparation procedures.^2^ This is mostly due to the fact that wet chemistry is the preferred way to tackle the analytical problem. The bias toward dilution is easily justified by the homogenization of the sample and the suppression of interferences, e.g., matrix effects, during the conditioning stages.^3^ When sample availability is limited, common questions arise regarding (i) which portions should be collected to yield representative results, (ii) what portion size is required to ensure the complete analytical routine can be performed, and (iii) how microdomains can be adequately characterized and reported, if possible at all. To address the aforementioned dilemma, laser ablation (LA) has been proposed as a highly efficient and straightforward solution.^4^ Acting directly on the solid and, thus, circumventing a large number of drawbacks tied to wet chemistry, the spatial resolution and flexibility achievable by LA methods are unmatched. The wide variety of parameters to be optimized in LA plays a key role in expanding the scenarios under which the technique can be applied.^5^ Although the initial need to tune parameters and the inherent complexity of the laser-matter interaction may discourage its use, contemporary literature has reached a high level of maturity regarding its applications to chemical analysis in combination with optical or mass detection, ranging from surface mapping^6,7^ to scalable nanoparticle synthesis.^8^

When combined with optical techniques, laser-induced breakdown spectroscopy (LIBS) is typically the first to come into play. The full compatibility of the instrumental layout required to perform both techniques grants high synergy between them, enabling the completion of successful studies even in extreme scenarios, such as single-particle characterization,^9^ remote analysis (including planetary exploration),^10^ or full bulk inspection within single events,^11^ to name a few examples. It should be noted that LIBS cannot occur without a previous LA event, since the phenomenon originates from the partial sublimation of the target when enough energy excess is conferred by the impinging excitation pulse. Over the years, LIBS has evolved into an outstanding tool capable of producing a high volume of data.^12^ Moreover, LIBS spectra can be rather easily fused to other simultaneously produced information sources such as Raman spectroscopy^13^ or plasma acoustics^14^ to expand the sample depiction. While, at first glance, working with a set featuring up to 10^6^ data points may appear daunting, paired with modern data processing schemes and classification tools, particularly those based on machine learning algorithms, LIBS has been proven as an efficient pathway toward accurate qualitative and quantitative sample representation, as evidenced from recent imaging^15^ and classification studies.^16,17^

Yet, on the issue of the safeguard of microdomains, thermal effects tied to LIBS when *ns*-pulse length, the most common due to instrumental simplicity and the yield of high raw signal, is used to ablate and excite the sample, may end up distorting the results. This drawback is further magnified by the need of multiple ablation events at the same spot to yield the final result. As an immediate solution, MS comes forward. Nonthermal ablation (using *ps- and fs-length pulses*) can generate sufficient material for LA-ICP-MS to perform successful characterization studies with high spatial resolution, even for delicate targets such as cells.^18^ Yet, the main inconvenience for ion detection is the need to transfer the ablated sample from the chamber in which the atomic vapor is produced into the mass spectrometer. This may cause losses along the transference path as well as distortions in the shape and composition of the originally produced material due to condensation.^19^ In addition, MS is a more demanding technique than optical sensing. Apart from instrumental costs, peripherals such as gases, infrastructure, or maintenance and expertise are higher than for ns-based spectroscopic setups. Therefore, an alternative method to conventional LIBS or MS is required when high signal, high spatial resolution, and negligible sample alteration are required, yet limited ablation events can be performed owing to the target’s scarcity.

Microanalysis has traditionally referred to the chemical interrogation of exceptionally small quantities of material. However, the real conceptual advance occurs when analytical strategies are designed to probe exclusively tiny and spatially restricted fractions of a heterogeneous sample.^20^ In this context, our group explored the combination of atmospheric optical trapping (OT) and LIBS (OT-LIBS) for the characterization of solids within the ablation chamber on a single particle (SP) basis by occluding and probing spheres produced after LA of the target.^21,22^ The proposed methodology, namely μLA-OT-SP-LIBS, is a two-step scheme that decouples the ablation process from plasma generation. Under this configuration, a single spot aerosol generation (SSAG) is created by a first laser shot (operating below the sample’s plasma formation threshold) to minimize damage to the sample while increasing spatial resolution. The generated fine cloud of particulate material is subsequently interrogated by LIBS inside the same sampling chamber. The two-step LIBS scheme ensures an excellent spatial resolution from the first laser shot, allowing for better chemical information to be extracted from the ablation process. In fact, the ablation of fine-generated particles leads to improve chemical characterization, as the particles are more readily isolated from matrix effects during plasma generation. Thus, μLA-OT-SP-LIBS is presented as an all-laser suite platform that combines laser ablation microsampling, optical catapulting, optical trapping, and LIBS for the chemical characterization of samples with a high degree of heterogeneity, which aligns with the principles of modern microanalysis.

In this work, we seek to validate the proposed methodology as a simultaneous multielemental microsampling technology. The capability of discerning and characterizing neighboring discrete domains within complex, valuable samples, even in the extreme scenario of using a single ablation pulse at each sampling site, is evaluated. Hence, we strive to achieve a successful characterization while ensuring virtually no target alteration and the required absolute sample amount in the range of low μg for a precise depiction of the sample. As a case study, we applied μLA-OT-SP-LIBS to a carbonaceous chondrite, a type of carbon-rich meteorite that contains chondrules, with the aim of distinguishing these geological domains from the surrounding rock matrix in which they are embedded, through inspection of single particles generated by a single ablation event.

## Experimental Section

### LIBS Experimental Setup

The methodology proposed for the single-particle analysis of meteorites is based on the OC-OT-LIBS platform described in previous studies.^23,24^ Particle generation followed the general procedure outlined in ref 21. Under the proposed workflow, particles were first generated in situ by LA within a chamber at atmospheric pressure. Subsequently, the particles were trapped on the flight by the trapping laser and the levitated particles were then analyzed by LIBS. All three steps were performed within the same sampling chamber, minimizing sample loss and preventing contamination.

Particles were generated via laser ablation of bulk meteorites. A coaxial line of view (CMOS camera; Thorlabs) was implemented to visualize the sample’s surface and to precisely select the area to be inspected. A schematic diagram of the μLA-OT-SP-LIBS instrument is illustrated in Figure 1. For reference, the meteorite sample was placed coincident with the optical trapping axis.^22^ After the alignment of the chosen area, the sample was ablated using a nanosecond laser pulse (Q-Switch Nd:YAG laser; 1064 nm; 5 ns pulse duration) directed through a series of mirrors into a microscope objective (ThorLabs, USA; magnification 10×; N.A.: 0.25), which focused the laser radiation on the target surface. After a single laser shot, the ablated material produced a solid aerosol that recirculated inside the sampling chamber. Then, an upward propagating CW Nd:YAG laser (532 nm; 180 mW at the beam focal region) was used to capture and isolate the particles at a steady position. Larger particles, after losing the initial momentum, were deposited at the bottom of the sampling chamber, whereas smaller particles recirculated inside the chamber due to Brownian motion for longer periods.^23^ Trapping was considered stable once no confined particle-aerosol collisions could be observed in our visualization system.

**Figure 1:**
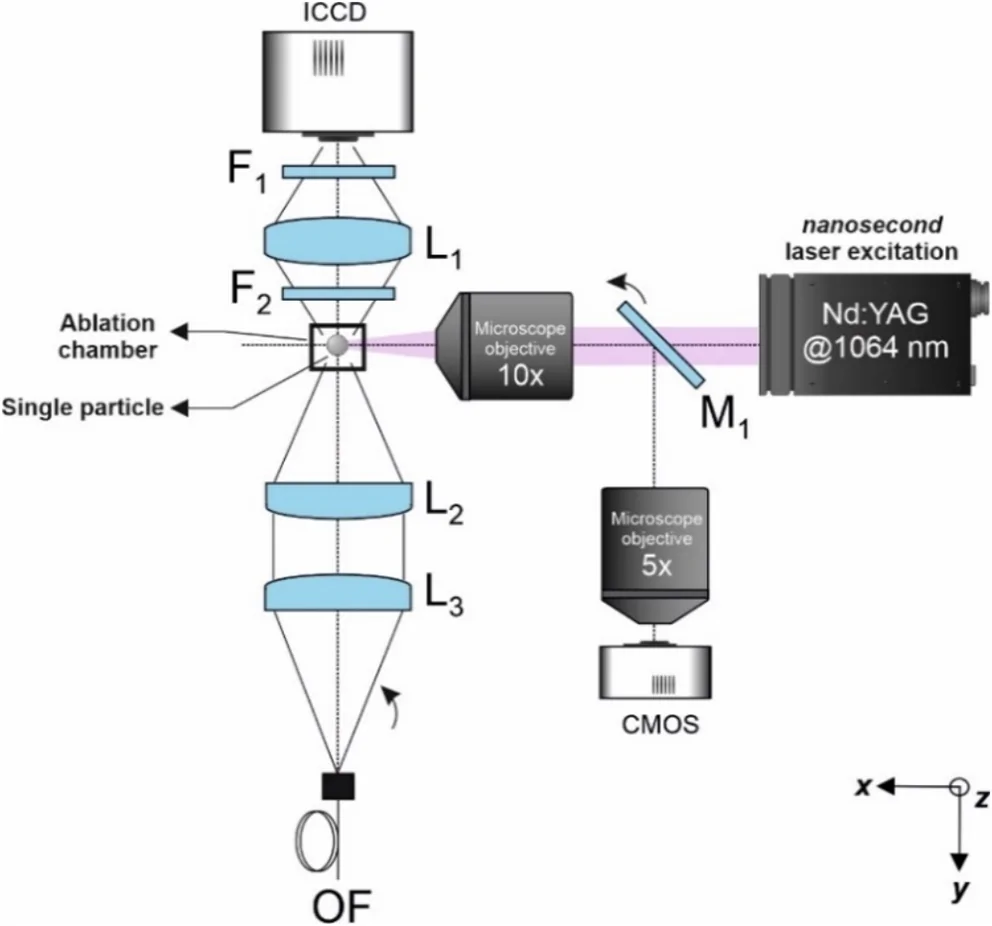
General view of the experimental setup used for μLA-OT-SP-LIBS in the *xy*-plane. Optical components include a flip mirror (M1), optical filters (F1 and F2), biconvex lens L1, and planoconvex lenses L2 and L3 used for plasma light collection. OF: trifurcated optical fiber.

Due to the Gaussian beam energy profile of the trapping laser, the number of particles trapped at once could vary from a single particle at the focus to a string of particles of different sizes along the beam waist. Out-of-focus trapping is linked to orders of magnitude less trapping force being exerted on the particles by the laser when compared to that experienced by spheres located at the focal region. Hence, contactless manipulation of these strings by switching the position of the trapping beam focus using a micrometric stage was enough to expel out-of-focus particles from the trap and keep at-focus single particles. The location of single-trapped particles was further manipulated to positioned them at the (0,0,0) coordinate to which all components of the instrumental system were aligned. The protocol for the multialignment scheme is described in ref 25. Briefly, a multistep routine based on an iCCD camera (AndorIstar DH334T, 2048 × 2048 pixels, Andor, UK) placed on the *y*-axis was developed to ensure precise alignment of every system component. Additionally, the same iCCD camera was used to track the light scattered by the individual particle along the trapping axis. Once the particle was stable, LIBS analysis was performed using the same laser that was used for laser ablation. To ensure that the position of the bulk sample did not interfere with the subsequent LIBS analysis, once the particles were generated, the sampling chamber was slightly displaced in the *x*-axis.^21^ The energy density was kept constant at 94 J cm^–2^.

Plasma emission was collected using a pair of planoconvex lenses (UV-FS; 2″ diameter; 100 mm focal length) that focused the plasma light to the entrance of a trifurcated optical fiber (Avantes; N.A.: 0.22; 600 μm diameter) connected to a time-integrated Czerny-Turner spectrometer (Avantes). A 1200 lines/mm holographic diffraction grating was employed to record light emission in the 200–800 nm spectral range. The lasers were externally synchronized using two pulse and delay generators, which allowed an exhaustive control of the interpulse delay and data-acquisition parameters. To minimize the spectral background while increasing the signal-to-noise ratio (SNR) of the selected spectral line, the acquisition delay time for LIBS analysis was kept constant at 5.28 μs. The integration window was fixed at 1.1 ms as given by the used spectrometer.

### Complementary Analytical Techniques for Sample Characterization

Alongside μLA-OT-SP-LIBS studies, other well-established techniques were used to inspect the generated particles in order to better contextualize the acquired results. For assessing morphology and elemental composition, scanning electron microscopy (SEM) was used in conjunction with energy-dispersive X-ray spectroscopy (EDX). X-ray diffraction (XRD) studies were used to elucidate the crystalline phases present in both inspected regions of the meteorite and provided further information regarding the physicochemical properties of the meteorite. Finally, X-ray fluoresecence (XRF) was used to obtain semiquatitative information on the bulk sample. The insight provided by X-ray techniques was of high relevance to understand the particularities of laser-matter coupling at the two probed areas and, in consenquence, the material produced by their LA.

- **Scanning electron microscopy–energy dispersive X-rays (SEM–EDX).** SEM micrographs were recorded to evaluate the morphology and size of the generated particles by laser ablation of the bulk meteorite sample. Particles produced after 10 laser pulses were collected on the surface of a glass slide placed perpendicular to the bulk meteorite sample and observed in a Dual Beam Field Emission Scanning Electron Microscope with a Focused Ion Beam (FESEM-FIB, Helios Nanolab 650), equipped with energy dispersive spectroscopy (EDS, Oxford Instruments). EDX results are given in Table S1.
- **X-ray Diffraction (XRD).** Transmission powder X-ray diffraction data using Mo Kα_1_ radiation were recorded using a Bruker D8 ADVANCE diffractometer. The measurement was performed from 3° to 45° (2θ) for 120 min. The tube was operated at 50 kV and 50 mA. The crystalline phases identified by XRD in the bulk meteorite are given in Table S2.
- **X-ray Fluorescence (XRF).** The bulk sample was analyzed in a helium environment using a wavelength-dispersive X-ray fluorescence spectrometer (ARL PERFORM ´X, Thermo Fisher Scientific). To perform XRF analysis, the sample was milled in an agate mortar and then dried at 105 °C for 2 h. XRF results are provided in Table S3, showing that Fe, Si, and Mg were the main components of the studied meteorite.
- **Elemental analysis**. The determination of the C, H, and N content in the sample was performed in a LECO TruSpec MACRO CHN elemental analyzer. The sample was milled in an agate mortar, and then, the elemental analysis was performed by total combustion of the sample at 950 °C in a controlled O_2_ atmosphere. The detectors are independent for each element, consisting of solid-state nondispersive IR (C, H) and differential thermoconductivity (TCD) for N.

### Meteorite Sample

Particles generated from the Jbilet Winselwan meteorite were individually analyzed in the *μLA-OT-SP-LIBS* system. This meteorite, illustrated in Figure 2, was purchased from a store specializing in the sale of fossils, minerals, and meteorites (www.litos.net). Jbilet Winselwan was classified in The Meteoritical Bulletin N° 102 of the Meteoritical Society as a CM-type carbonaceous chondrite.^26^ The meteorite contains chondrules of Types I and calcium–aluminum-rich inclusions (CAIs) within an iron-based matrix. The chondrule/matrix ratio is ∼50:50, with an average chondrule size of 200 μm (up to 1.2 mm) and a relatively small size for CAIs (below 800 μm). Major silicate compositions are olivine (fayalite and forsterite) and pyroxenes.

**Figure 2:**
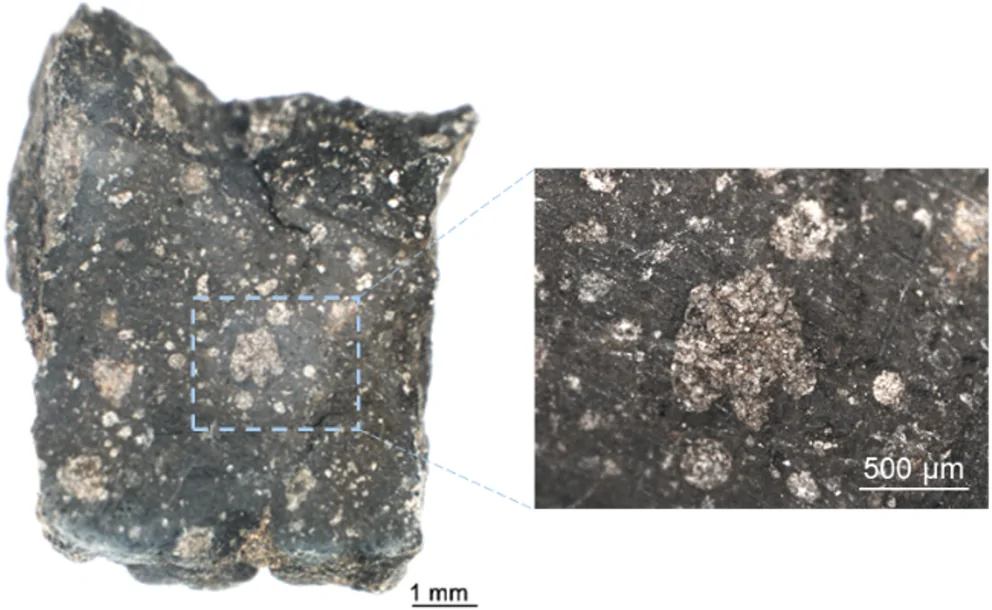
Bulk fragment of the Jbilet Winselwan meteorite. The main image shows the overall texture of the specimen (scale bar = 1 mm). The inset presents a photographic detail of chondrules embedded in the fine-grained matrix (scale bar = 500 μm).

### Sample Preparation

The procedure for sample preparation, adjusted to the specific requirements of the experiment, involved cutting the samples to a 3 mm thickness using a precision cutting machine (Minitom, Struers) operated at low speed with distilled water as a coolant. The prepared meteorite fragment was then attached to the inner wall of a quartz cuvette with a 1 cm optical path. The cuvette was sealed using a 200 μm thick glass slide. To prevent contamination, gloves were used while handling the samples and the sampling chamber.

## Results and Discussion

The aim of this study was to evaluate the potential of laser ablation microsampling combined with optical trapping and single-particle LIBS (μLA-OT-SP-LIBS) for the chemical characterization of discrete microdomains within heterogeneous meteoritic material. This methodology was applied to the carbonaceous chondrite Jbilet Winselwan, a meteorite with a markedly heterogeneous structure composed of a Fe-rich matrix enclosing numerous chondrules of variable size and texture. Through controlled ablation events, particles were selectively generated from both a representative chondrule and the surrounding matrix, then trapped in the air and analyzed individually by LIBS. To place these findings in a broader mineralogical and elemental context, additional data sets were acquired. SEM-EDX was employed on representative particles to examine their morphology and major-element chemistry, while bulk mineral phases and elemental abundances of the meteorite were characterized by XRD and XRF, respectively. Bulk characterization confirms the compositional contrast between the Fe-rich matrix and the more Mg- and Na-enriched chondrule domains. SEM-EDX shows higher Mg, Al, and Na contents in the chondrule, whereas the matrix exhibits substantially greater Fe and Ca abundances. XRD analysis reveals a mineral assemblage dominated by forsterite, enstatite, albite, and serpentine-group phases, consistent with the petrology of CM-type carbonaceous chondrites. Complementary XRF measurements further support this framework, indicating a bulk composition enriched in Fe- and Si-bearing oxides with significant MgO and minor contributions from alkali and transition-metal oxides. These complementary results, summarized in the Supporting Information, provide the structural and chemical framework for interpreting the single-particle LIBS data presented below.

### In Situ Particle Production in the Carbonaceous Chondrite Meteorite

Prior to OT and subsequent LIBS characterization, chondrule and matrix particles were generated by laser ablation of the meteorite using 10 laser pulses at 79 mJ/pulse. Figure 3 illustrates the schematic diagram for particle production in μLA-OT-SP-LIBS. The material ejected from the ablated volume produced a polydisperse solid aerosol. To study the particle morphology, the material deposited at the bottom of the cuvette after aerosol full relaxation was evaluated by SEM.

**Figure 3:**
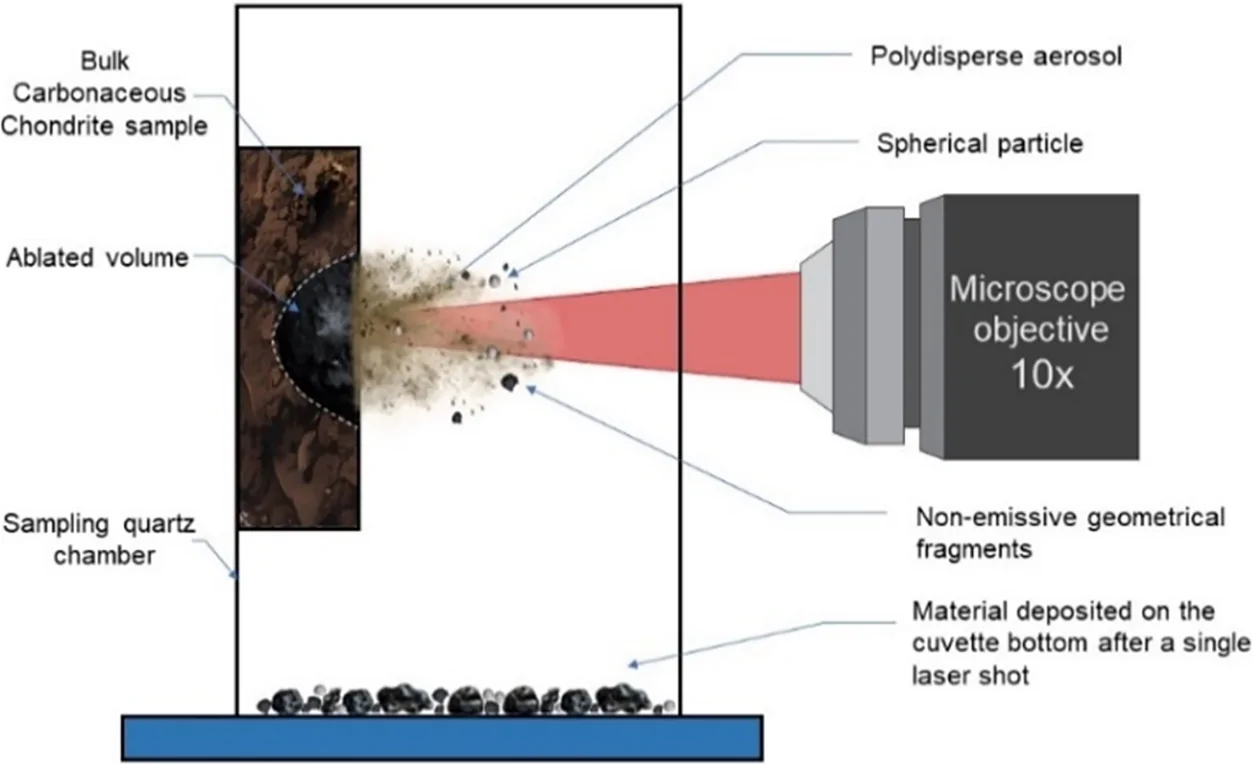
Schematic diagram of particle generation in the sampling chamber during μLA-OT-SP-LIBS.

SEM images are shown in Figure 4. Three main types of particles were recognized: spherical particles (Figure 4A), dendrite-structured agglomerates (Figure 4B), and nongeometrical massive fragments (Figure 4C). The spherical particles were derived from the direct ejection of molten droplets that solidified instantly.^21^ These constituted the particles that were selectively trapped in our system. In addition, due to the condensation of vaporized matter after laser ablation, particle agglomerates with dendritic structures were also formed. However, the irregular shapes of the dendrite-structured agglomerates led to multiple scattering points, preventing steady trapping. It must be stressed that optical trapping relies on gradient forces that push the isolated particle toward the light intensity maximum at the center of the trap. Gradient forces are opposed by scattering forces, arising from momentum transference from the impinging CW laser’s photons to the trapped entity and pushing it out of the optical trap. Lastly, nongeometrical fragments showed a behavior similar to that of the agglomerates, with their trapping being prevented due to their morphology and size. The most plausible origin of these fragments is the fracture of small layers of the sample surface under the pressure exerted by the shockwave generated during the early stages of the plasma onset.^27^ Such specimens likely imply that the focus of the ablation laser was set slightly below the surface of the target during the studies reported herein. Therefore, discrete spherical particles were the only particles that could be successfully analyzed in the employed platform.

**Figure 4:**
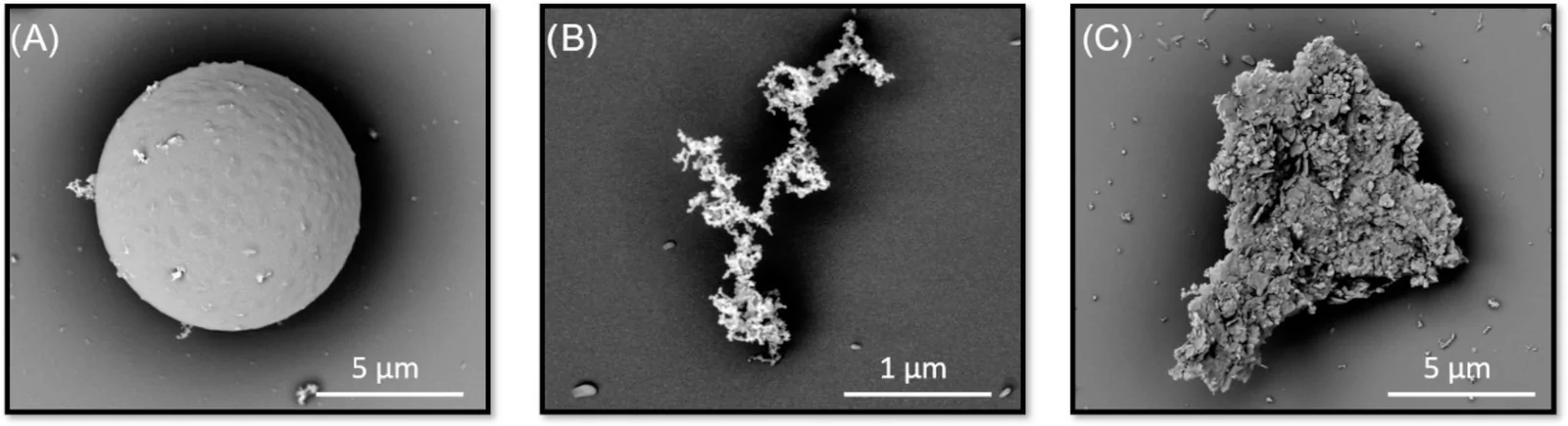
SEM images of three main types of particles generated by laser ablation: (A) Spherical particles, (B) dendrite-like agglomerates, (C) nongeometrical and nonemissive fragments.

To estimate the number of particles generated by a single laser pulse, the crater depth (in both the iron-based matrix and the chondrule) at a fixed laser energy of 79 mJ/pulse was examined using profilometry. The results are plotted in Figure 5. Crater depths were quite similar, 100 and 120 μm, for the chondrule and the Fe-based matrix, respectively. Taking into account the crater size, the total ablated volume was estimated, considering a conical geometry, at approximately 3.7·10^6^ μm^3^ (chondrule) and 6.4·10^6^ μm^3^ (Fe-based matrix). Particles produced were examined from SEM micrographs acquired at 1000× magnification. An image-processing analysis scheme based on SEM data was developed using the StarDist plugin in Fiji.^28,29^ The StarDist plugin allowed the identification of the particles in the image and also to determine parameters such as the total number of particles, the size, and the sphericity degree (expressed as the ratio between the minor and major axes of each individual particle, δ). For coherence with the morphological restriction imposed by the optical trap, quantification studies were performed only on particles exhibiting a sphericity degree above 0.7. Table 1 summarizes the calculated parameters using the aforementioned data processing scheme. It is worth noting that, while the total number of particles and the density (particles/mm^2^) are considerably higher in those particles generated in the Fe-based matrix, the percentage of particles presenting δ > 0.7 is larger for those generated in the chondrule. The average particle diameter, applying the δ > 0.7 criteria, was calculated in 1.30 μm for the Fe-based matrix and 1.46 μm for chondrule-originated particles. Moreover, the calculated standard deviation for both sampling sites revealed that the aerosol exhibits a low-level polydispersity.

**Figure 5:**
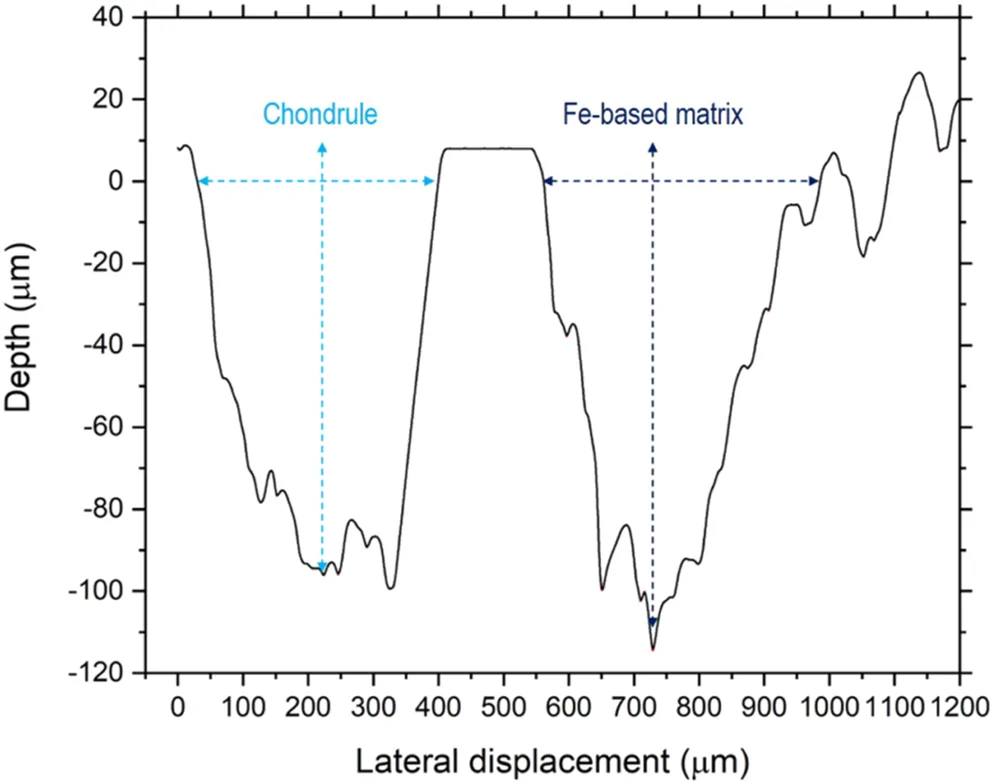
Depth profile craters after a single laser pulse across a chondrule and Fe-based matrix in the Jbilet Winselwan meteorite, obtained by profilometry.

**Table 1:** Parameters Obtained from SEM Images Using the StarDist Plugin for Fiji

	matrix	chondrule
Total number particles	4731	2563
Number of particles δ > 0.7	3427	2059
% δ > 0.7	72	80
Particle diameter δ > 0.7 (μm)	1.31	1.46
*s*	0.05	0.21
Particles/mm^2^	22,305	9707

The response of solids to laser ablation is closely related to both laser parameters and solid properties/structures.^30^ According to the results obtained, laser ablation appears to be more efficient in the fine-grained matrix than in the chondrules, leading to the production of a larger number of spherical particles. This behavior can be attributed to microstructural and compositional differences. The matrix consists mainly of very fine silicates, sulfides, and amorphous material,^31,32^ which absorb the laser energy more effectively and restrict heat dissipation, promoting rapid melting and vaporization.^30^ Some authors designate the matrix as a low-temperature component. In contrast, chondrules are considered high-temperature components,^33^ mainly dominated by well-crystallized olivine and pyroxene, whose ordered lattices and higher thermal conductivity limit localized heating. Even though the ablated volume was only ∼1.7 times larger in the matrix than in the chondrule, a nearly 2-fold number of particles was produced, consistent with the intense, localized “explosive boiling” reported for amorphous targets under nanosecond-pulse irradiation.^30^ Together, these factors make the matrix more prone to efficient ablation and to the generation of molten droplets that solidify as the observed spherical particles.

### LIBS Analysis of Single Meteorite Particles

Figure 6A depicts the areas selected for laser ablation, whereas Figure 6B presents the SEM images of the spherical particles generated. For LIBS analysis, 15 and 20 SP-LIBS spectra in the 200–800 nm spectral range were recorded in the chondrule and the Fe-based matrix, respectively. It must be noted that the chondrule selected to generate the particles was 550 μm in diameter, a size larger than the laser spot size of 350 μm, ensuring that the produced particles were exclusively from that chondrule.

**Figure 6:**
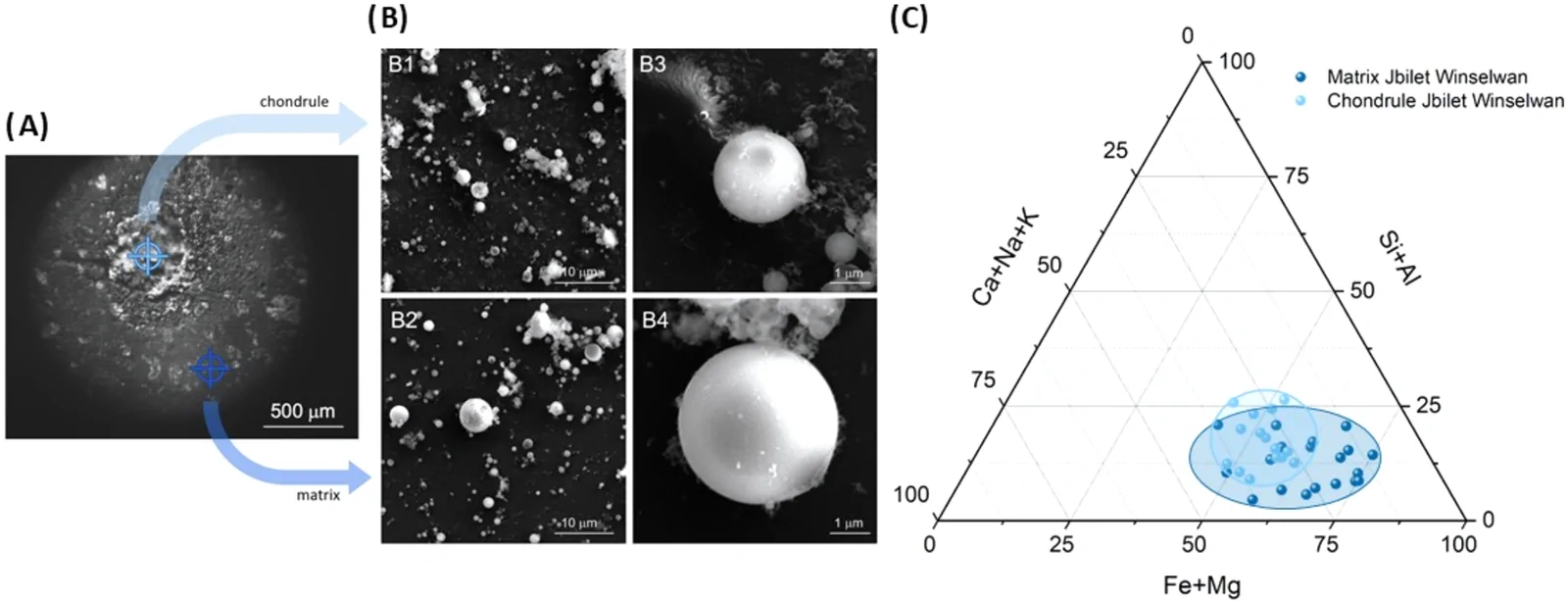
(A) Image (obtained with the visualization system of the OT-LIBS platform) of the areas of a chondrule and matrix of the carbonaceous chondrite where the particles have been generated. (B1–B2) Panoramic SEM images of the particles generated in both areas of the meteorite. (B3–B4) Detail of the spherical particles acquired by SEM of both the chondrule and the meteorite matrix. (C) Ternary diagram representing the Si+Al intensities; Ca + Na + K and Fe + Mg from individual particles of the matrix and chondrule of the Jbilet Winselwan meteorite.

The emission lines of 396.18 nm for Al (I), 396.88 nm for Ca­(II), 274.89 nm for Fe­(I), 285.14 nm for Mg­(I), 766.49 nm for K­(I), 589.55 nm for Na­(I), and 288.13 nm for Si­(I) were used for data processing, as they provide the strongest signal and allow reliable identification and quantification of each element in SP-LIBS events. With the purpose of discriminating the individual particles of the chondrule and the matrix, Figure 6C shows a ternary diagram based on the LIBS intensity of Si + Al, Ca + Na + K, and Fe + Mg. This representation allows to differentiate the meteorite particles based on their chemical composition (absence or presence of minerals such as pyroxenes, olivines, and feldspars), thus providing very valuable information from a geochemical point of view. As shown in the graph, chondrule particles were well grouped, whereas those from the Fe-based matrix were dispersed, indicating two distinct contributions.

LIBS analysis was also employed to probe the compositional heterogeneity of the meteorite through differences in elemental intensity ratios between the matrix and chondrule domains. Figure 7A plots the binary Na/Fe–Mg/Fe diagram. In this graph, the particles from the matrix were better grouped. These matrix particles exhibited lower Mg/Fe and Na/Fe ratios than those from the chondrule. The higher Mg/Fe ratios in the chondrules reflect their enrichment in forsterite (olivine) and enstatite (pyroxene),^31^ leading to greater magnesium and lower iron contents compared to the matrix, as confirmed by EDX analysis (Table S1). The elevated Na/Fe ratios suggested that the albite was likely present within the chondrules. These mineral phases were detected through XRD (Table S2).

**Figure 7:**
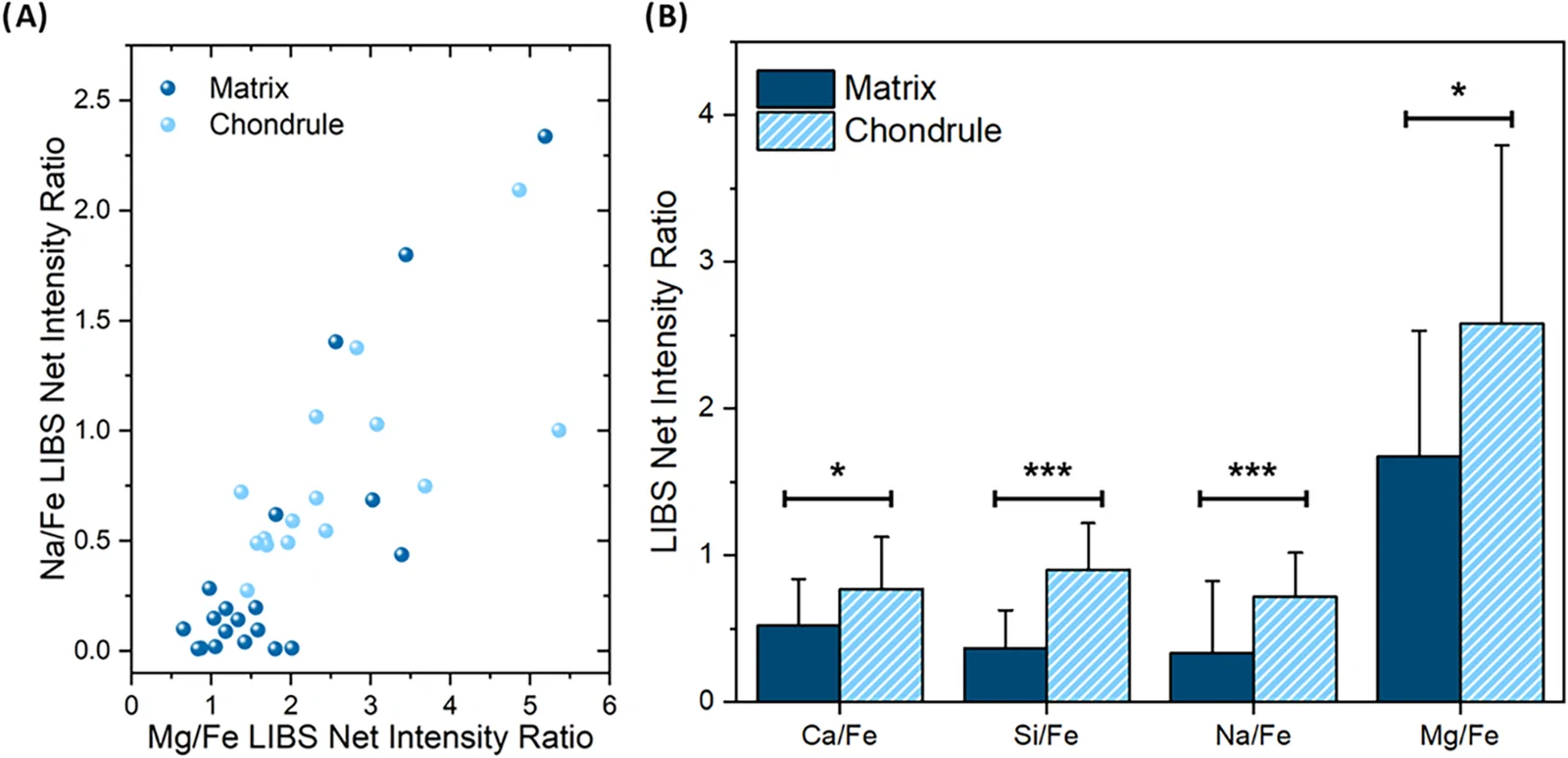
(A) Binary diagram of the intensity ratios Na/Fe and Mg/Fe obtained by LIBS for the generated particles in the chondrule and the iron-based matrix of the Jbilet Winselwan meteorite. (B) Net intensity ratios for the major emission lines featured in the recorded SP spectra. Grubb’s test was performed to eliminate outliers (a maximum of 1 data point was allowed to be eliminated per set) prior to a Levene’s test for variance comparison. The significance test used for the paired samples was either a standard two-sample *t* test (equal variance) or a Welch’s *t* test (unequal variance). Data sets used in the figure consisted of *n* = 19 and *n* = 15 points for matrix and chondrule particles, respectively. Error bars represent the standard deviation of the mean value. The asteriscs indicate the *p*-values: *: < 0.05; ***: < 0.001.

Moreover, some matrix particles presented a composition akin to that of the chondrule, which may indicate that they may have been derived from smaller-sized chondrules occluded within the ablated area of the matrix. Regarding spectral lines, particles produced at each of the ablated meteorite areas exhibited quasi-identical features upon qualitative inspection. Hence, significant differences were observed in the major elements/Fe intensity ratios (Figure 7B). Iron was chosen as a reference element, as it is the main component of the rock matrix, as confirmed by semiquantitative XRF analysis (Table S3). The lower Fe content of the chondrule resulted in higher ratios, which, despite the relatively high standard deviation, as expected from SP-LIBS,^23^ allowed the statistical differentiation of the particles’ origin. Furthermore, since multielemental spectra are acquired in μLA-OT-SP-LIBS, simple statistical tools such as two-sample *t* tests can be used to classify particles, as the decision is based on several criteria, i.e., multiple different ratios. It is worth mentioning that higher Si/Fe, Na/Fe, and Mg/Fe ratios in particles from the chondrule are in good agreement with EDX results. In view of the results acquired from the single spheres analyzed and the feasibility of attributing them to their geological domain of origin, μLA-OT-SP-LIBS may be confirmed as a highly sensitive tool for the characterization of exiguous sample quantities, down to the particulate material produced by a single ablation pulse, hence requiring negligible sample damage.

### Searching for Organic Matter in Particles of Jbilet Winselwan

Carbonaceous chondrites are among the most primitive types of meteorites, and their study provides evidence of the early stages of the Solar System. These meteorites tend to contain 1–4 wt % organic matter, including carboxylic acids, aliphatic and aromatic hydrocarbons, and amino acids.^34^ Precisely, these organic compounds have led to speculation that carbonaceous chondrites may have provided the necessary ingredients for the origin of life on Earth.^35^ Given the astrobiological interest of these molecules and to further expand the capabilities of μLA-OT-SP-LIBS, a thorough examination of the individual particles of the Jbilet Winselman meteorite was conducted to ascertain the presence of any potential organic signals in the LIBS spectra. In this regard, the CN band systems are recognized as the primary molecular emissions for the identification of organic samples in LIBS-based inspection. Figure 8 shows the emission spectra of three individual particles from the matrix and a representative example of SP spectra of chondrule-originated particles, in the wavelength range corresponding to the main CN molecular band from the Violet System (*B* ^2^∑^+^ – *X* ^2^∑^+^, Δν = 0), λ = 385–389 nm. To facilitate identification of molecular features, emission from an organic standard was included to highlight the band’s main vibrational transitions. No organic signal could be observed in the chondrule. On the other hand, in the matrix, the CN transitions (1,1) at 387.14 nm and (0,0) at 388.34 nm were detected in three of the 20 particles analyzed, thus allowing identification of the molecular signal. The remaining vibrational modes of the emission band, namely (2,2), (3,3), and (4,4), were probably interfered by the contribution of N_2_, N_2_^+^ and N, also present in the same wavelength window.^25^ In single-particle LIBS, the concomitant ionization of the air surrounding the particle is the primary component of the laser-induced plasma during plasma expansion.^36,37^ Consequently, CN formation in SP-LIBS primarily occurs via the combination of carbon atoms ablated from the sample with free nitrogen atoms dissociated from atmospheric N_2_ molecules. These emissions predominantly originate from the recombination of native atomic carbon in the plasma with atmospheric nitrogen. The reported sensitivity of single-particle LIBS for C in graphite particles (75 fg for particles of 400 nm in diameter)^25^ indicates that carbon, if present in the sample, could be readily detected by this technique. Considering the background literature, the exclusive presence of CN signatures in matrix-derived spectra suggests that the detected carbon is native to the sample.

**Figure 8:**
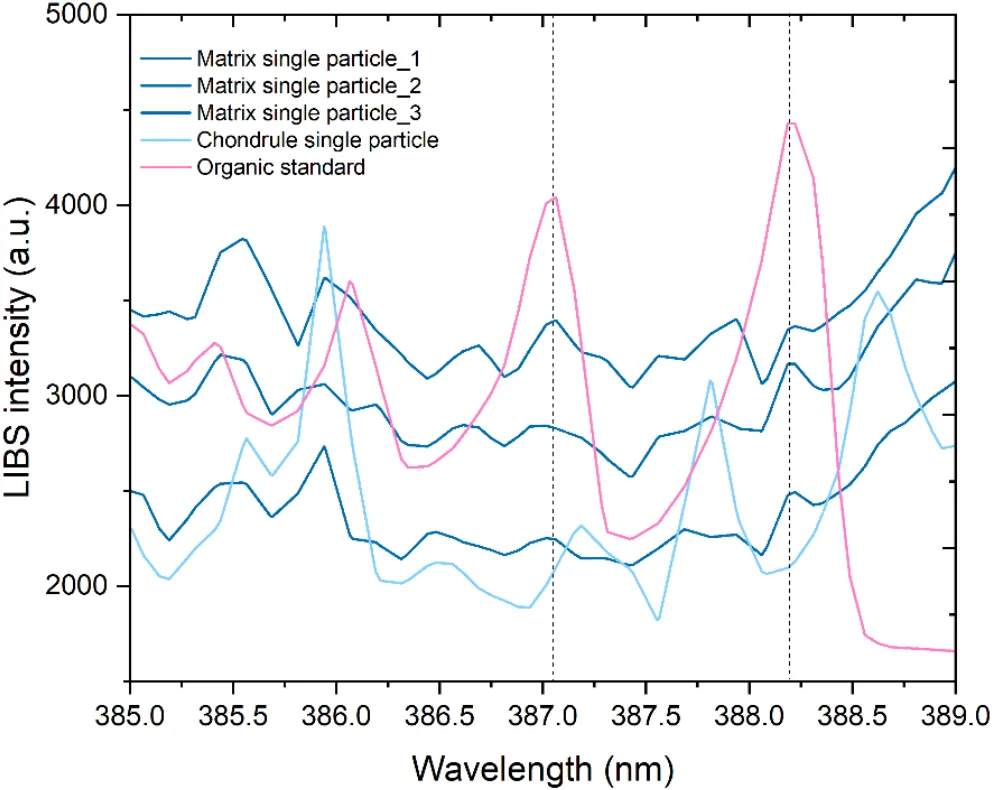
CN signal at 387.06 and 388.19 nm from three individual particles from the Jbilet Winselwan matrix. To better identify the signal from the CN band, the LIBS spectrum of an organic standard is also shown in the graph.

To confirm the origin of this molecular signal, the sample was subjected to total combustion to determine the elemental composition of C, N, and H. The total organic fraction was obtained by subtracting the total inorganic fraction from the total measured value. To determine the inorganic fraction, the sample was previously calcined at 550 °C for 6 h. The results are summarized in Table 2. The carbon content (CT) was found to correspond almost exclusively to the organic fraction (nearly 1.8% w/w). Conversely, organic nitrogen (NO) concentration was close to the limit of detection (around 0.1%) while the hydrogen content (HT) was ca. 1%. This low content suggests that a secondary pathway for CN formation via combination with this native nitrogen was unavailable. These results hint the potential use of μLA-OT-SP-LIBS for the detection of organic signatures by microsampling geological targets. This information may motivate further studies using LIBS or other complementary techniques to deepen the understanding of such signatures and justify the use of more aggressive sample treatment protocols.

**Table 2:** Composition in Weight Percent of Chondrite Matrix as Measured by Elemental Analysis

Sample ID	% TC^a^	% TIC^b^	% TOC^c^	% TH^d^	% TN^e^	% TIN^f^	% TON^g^
Chondrite	1.84	0.14	1.72	1.00	0.15	0.06	0.09

a Total carbon.

b Total inorganic carbon.

c Total organic carbon.

d Total hydrogen.

e Total nitrogen.

f Total inorganic nitrogen.

g Total organic nitrogen.

## Conclusions

This work establishes laser ablation microsampling coupled with optical trapping and single-particle laser-induced breakdown spectroscopy (μLA-OT-SP-LIBS) as a highly sensitive and minimally invasive analytical strategy for the chemical characterization of scarce and compositionally heterogeneous materials. By combining controlled nanosecond laser ablation with in situ particle trapping and single-particle optical emission analysis, the method enables multielemental and molecular assessment of discrete microdomains from a total sampled mass in the low microgram range. Using the Jbilet Winselwan carbonaceous chondrite as a benchmark specimen, the approach successfully discriminated between particles derived from Fe-rich matrix regions and those originating from silicate chondrules based on elemental intensity ratios, particularly Mg/Fe and Na/Fe. These results were fully consistent with complementary bulk analyses (XRD, XRF, SEM-EDX), demonstrating that the microsampling protocol yields representative chemical information even under extreme mass-limited conditions.

The single-particle LIBS spectra obtained from trapped micrometric particles (1–2 μm) also revealed CN molecular emissions in selected matrix-derived particles, indicating the presence of native organic carbon. This finding is consistent with the known occurrence of prebiotic carbonaceous matter in CM-type meteorites and underscores the potential of μLA-OT-SP-LIBS for detecting molecular species in extraterrestrial or astrobiologically relevant samples. The ability to identify organic signatures within individual particles while preserving the integrity of the parent material represents a significant advance over conventional analytical workflows requiring extensive sampling and preparation.

Overall, μLA-OT-SP-LIBS integrates the spatial precision of laser ablation, the selectivity of optical trapping, and the spectroscopic versatility of LIBS within a single analytical platform. This method enables rapid, contamination-free, and minimally destructive analysis of multicomponent microdomains, requiring only negligible amounts of material. Its demonstrated capability for simultaneous elemental and molecular characterization expands the analytical possibilities for rare geological specimens, microinclusions, and other valuable samples where sample preservation is essential.

## Supplementary Material



## Notes

Table comparing the different matrix and chondrule atomic composition (in w/w) obtained by EDX, table detailing the crystalline phases identified by XRD in the bulk meteorite, table detailing the composition of the meteorite (in w/w) obtained by XRF (PDF)
