# Supplementary material for: All-Laser Suite Analytical Platform (Microsampling, Catapulting, Trapping, and LIBS) for the Characterization of Discrete Domains in Samples of Astrochemical Interest
Source: Anal Chem. 2026 Aug 28;98(36):26465–73. doi: 10.1021/acs.analchem.6c01976 (PMC13584628; doi:10.1021/acs.analchem.6c01976)
Supplement: Supplementary file 1 [file ac6c01976_si_001.pdf]

Supplementary Information

ALL-LASER SUITE ANALYTICAL PLATFORM (MICROSAMPLING, CATAPULTING, TRAPPING AND LIBS) FOR THE CHARACTERIZATION OF DISCRETE DOMAINS IN SAMPLES OF ASTROCHEMICAL INTEREST

Clara Burgos-Palop[a,b], Tomás Delgado[a,b], Pablo Purohit[c], Francisco J. Fortes[a,b*], and José M. Vadillo[a,b*]

[a] UMALASERLAB, Departamento de Química Analítica, Universidad de Málaga, 29071 Málaga, España (Spain)

[b] Instituto de Materiales y Nanotecnología (IMANA), Facultad de Ciencias, Universidad de Málaga, 29071 Málaga, España (Spain)

[c] Universidad Complutense, Facultad de Químicas, Departamento de Química Analítica, 28040 Madrid, España (Spain)

*Corresponding author: F.J. Fortes (javierfortes@uma.es), J.M. Vadillo (jmvadillo@uma.es)

The supplementary information included provides information about the different elemental composition of the matrix and chondrule of the meteorite obtained by SEM-EDX, identification of the crystalline phases in the bulk meteorite by XRD, and semiquantitative analysis of the meteorite by XRF.

Table S1. EDX results %(w/w)

Element	Matrix	Chondrule
O	38.71	48.05
Fe	24.65	13.71
Si	20.20	20.49
Mg	8.66	10.53
Al	2.28	3.11
Ca	3.01	0.83
Na	1.17	1.98
K	0.49	0.91
Ti	0.68	0.39
Cr	0.09	-
**Total**	100	100

Table S2. Crystalline phases identified by XRD in the bulk meteorite.

Meteorite	Crystalline phases detected by XRD
Jbilet Winselwan	Forsterite: $(Mg_{0.903}Fe_{0.097})$
	$(Mg_{0.897}Fe_{0.103})(SiO_4)$
	Enstatite: $Mg(SiO_3)$
	Albite: $NaAlSi_3O_8$
	Lizardite (serpentine):
	$Mg_{2.74}Fe_{0.16}Al_{0.16}Si_{1.93}O_5(OH)_4$
	Serpentine: $H(Mg_2SiO_4)_8$

Table S3. Semiquantitative analysis in %(w/w) by XRF.

Compound	Jbilet Winselwan (weight %)
SiO_2	27.20
Fe_2O_3	36.23
MgO	15.74
SO_3	2.54
Al_2O_3	1.86
Na_2O	0.67
CaO	1.50
NiO	1.80
Cr_2O_3	0.47
MnO	0.26
P_2O_5	0.17
K_2O	0.06
TiO_2	0.10
Co_3O_4	0.04
CuO	0.02
ZnO	0.02
SrO	-
C,H,O,N	11.32
